# Africa-wide meta-analysis on the prevalence and distribution of human cystic echinococcosis and canine *Echinococcus granulosus* infections

**DOI:** 10.1186/s13071-022-05474-6

**Published:** 2022-10-05

**Authors:** Solomon Ngutor Karshima, Musa Isiyaku Ahmed, Nuhu Bala Adamu, Abdullahi Alhaji Magaji, Musa Zakariah, Konto Mohammed

**Affiliations:** 1 Department of Veterinary Public Health and Preventive Medicine, Federal University of Agriculture, PMB 28, Zuru, Kebbi State Nigeria; 2 Department of Veterinary Parasitology and Entomology, Federal University of Agriculture, PMB 28, Zuru, Kebbi State Nigeria; 3 Department of Veterinary Anatomy, Federal University of Agriculture, PMB 28, Zuru, Kebbi State Nigeria

**Keywords:** Africa, Dog, *Echinococcus granulosus* infections, Human cystic echinococcosis, Public health, Zoonosis

## Abstract

**Background:**

Echinococcosis is a neglected zoonosis of increasing public health concern worldwide. According to the World Health Organization, 19,300 lives and 871,000 disability-adjusted life-years are lost globally each year because of cystic echinococcosis. Annual costs associated with cystic echinococcosis were estimated at US$ 3 billion because of treatment of cases and losses in the livestock industry.

**Methods:**

We performed the random-effects model of meta-analysis using 51-year (1970–2021) data available from AJOL, Google Scholar, PubMed, Science Direct, Scopus and Web of Science. We also applied the Joanna Briggs Institute critical appraisal instrument for studies reporting prevalence data, the Cochran’s *Q*-test, Egger’s regression test and the single study deletion technique to respectively examine within-study bias, heterogeneity, across-study bias and sensitivity.

**Results:**

Thirty-nine eligible studies on human cystic echinococcosis (HCE) from 13 countries across the five African sub-regions showed an overall prevalence of 1.7% (95% CI 1.1, 2.6) with a statistically significant (*P* < 0.001) sub-group range of 0.0% (95% CI 0.0, 14.1) to 11.0% (95% CI 7.6, 15.7). Highest prevalences were observed in Eastern Africa (2.7%; 95% CI 1.4, 5.4) by sub-region and Sudan (49.6%; 95% 41.2, 58.1) by country. Another set of 42 studies on *Echinococcus granulosus* infections (EGI) in dogs from 14 countries across the five African sub-regions revealed an overall prevalence of 16.9% (95% CI 12.7, 22.3) with a significant (*P* < 0.001) variation of 0.4 (95% CI 0.0, 5.9) to 35.8% (95% CI 25.4, 47.8) across sub-groups. Highest prevalences of *E. granulosus* were observed in North Africa (25.6%; 95% CI 20.4, 31.6) by sub-region and Libya (9.2%; 95% CI 5.7, 13.9) by country.

**Conclusion:**

Human cystic echinococcosis and EGI are respectively prevalent among Africans and African dogs. We recommend a holistic control approach that targets humans, livestock, dogs and the environment, which all play roles in disease transmission. This approach should involve strategic use of anthelminthics in animals, standardized veterinary meat inspection in abattoirs, control of stray dogs to reduce environmental contamination and proper environmental sanitation. Mass screening of humans in hyper-endemic regions will also encourage early detection and treatment.

**Graphical abstract:**

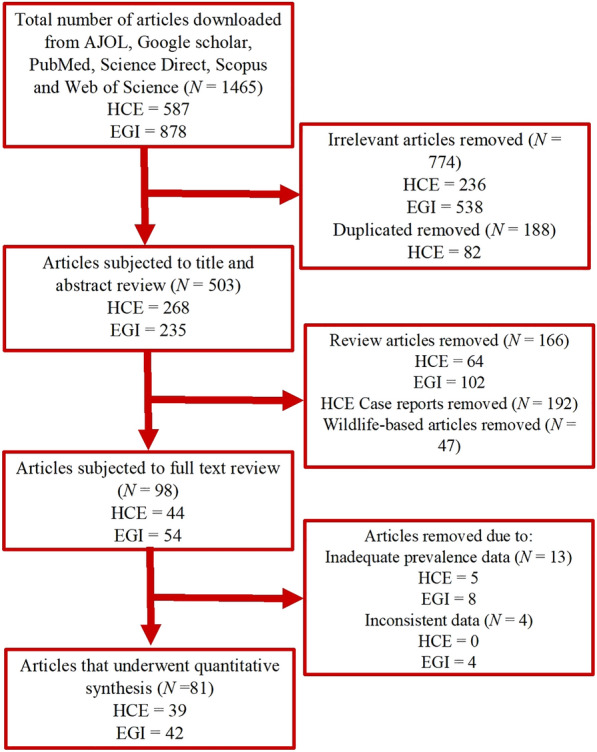

**Supplementary Information:**

The online version contains supplementary material available at 10.1186/s13071-022-05474-6.

## Background

Cystic echinococcosis is a cestodal zoonosis caused by metacestode of the tapeworm *Echinococcus granulosus*. *Echinococcus granulosus* (s.l.) is a complex comprising ten genospecies (G1–G9 and the lion strain). The genospecies G1–G3 are closely related and are referred to as *E. granulosuss* (s.s.) [1, 2]. The genospecies G1 (which is the common sheep strain) is the most common cause of human cystic echinococcosis (HCE) worldwide [3]. However, other zoonotic genospecies include strain G5 (*E. ortleppi*) and strain G6–G9, which are referred to as *E. canadensis* [4].

Cystic echinococcosis is classified among neglected zoonotic diseases by the World Health Organization [5] and is one of the helminthic diseases with the widest geographic distribution, existing in all continents of the world, with the exception of only Antarctica [6–8]. According to the estimations made by the foodborne disease burden epidemiology reference group of the World Health Organization, 19,300 lives and 871,000 disability-adjusted life-years are lost globally each year due to cystic echinococcosis. Annual costs associated with cystic echinococcosis were also estimated at US$ 3 billion because of treatment of cases and losses in the livestock industry [9].

The tapeworm *Echinococcus granulosus* infects the small intestine of canids, which serve as its definitive hosts. Herbivores act as intermediate hosts, where the parasitic larva, called hydatid cyst, causes a condition referred to as cystic echinococcosis. Humans acquire infection through the ingestion of food (particularly vegetable and water) contaminated with feces of infected dogs [10]. Humans do not contribute to the sustenance of the life cycle, hence are considered aberrant intermediate hosts or dead-end hosts [11]. *E. granulosus* is principally maintained in a domestic dog-sheep-dog cycle, where it is transmitted between stray or owned dogs and a number of domestic ruminant species [9].

The life cycle is complex, involving two hosts and a free-living egg stage. The dynamics of the transmission of the parasite are determined by the interaction of factors associated with these two hosts and with the external environment. Some of the factors that perpetuate cystic echinococcosis in humans may include farming activities involving livestock and dogs as well as home-slaughtering practices and dogs scavenging within abattoir premises [12].

Cystic echinococcosis usually remains asymptomatic for years before the hydatid cysts grow large enough to cause symptoms. Clinical symptoms are dependent on organs affected, cyst location within the organ, cyst size and the genotype of the parasite associated with infection [13, 14]. Symptoms may be associated with complications such as cyst rupture with resultant infection and anaphylaxis, fistula development with adjacent structures like biliary tract, intestine and bronchus and mass effects on neighboring structures [15].

Cystic echinococcosis is a zoonosis of increasing public health concern worldwide. Several individual surveillance studies on both HCE and *E. granulosus* infections in dogs (the definitive hosts) have been reported across the African continent. However, harmonized data on the pathogen in Africa are lacking. In this study, we performed a systematic review and meta-analysis of data published on cystic echinococcosis in humans and *E. granulosus* infections in dogs on the African continent between January 1, 1970, and December 31, 2021, and presented in this report Africa-wide prevalence and distribution of echinococcosis.

## Methods

### Study protocol

This Africa-wide systematic review and meta-analysis was performed in line with the 27 items recommendations of the Preferred Reporting Items for Systematic Reviews and Meta-Analyses (PRISMA) published by Moher et al. [16]. The basic requirement for inclusion of a study was the occurrence of cystic echinococcosis in humans and the infection of dogs with *E. granulosus.* We registered the review protocol on PROSPERO with registration number CRD42020208975 and available from: https://www.crd.york.ac.uk/prospero/display_record.php?ID=CRD42020208975.

### Information sources and search strategy

Authors systematically searched AJOL, Google scholar, PubMed, Science Direct, Scopus and Web of Science for a period of 6 months (1 October 2021 to 31 March 2022) for information published on HCE and *E. granulosus* infections in dogs from 1 January 1970 until 31 December 2021. Two different *MeSH* search strings were used in the present study: (i) “Cystic echinococcosis” OR “Hydatid cyst” OR “Metacestode of *E. granulosus*” AND “Humans” OR “Man” AND “Africa” and (ii) “*Echinococcus granulosus*” OR “*E. granulosus*” OR “Dog tapeworm” OR “Hydatid worm” OR “Hyper tapeworm” AND “Dogs” OR “Canids” OR “Carnivores” AND “Africa.” Additional studies were obtained through screening of citation lists and contact of authors and editors of journals for studies with inadequate information online.

### Study selection, data extraction and reliability

We screened the title of each downloaded article, followed by its abstract for relevance. Thereafter, studies that were apparently relevant were subjected to full text review for extraction of data using the inclusion criteria. Criteria for inclusion of a study was that it: (i) investigated cystic echinococcosis in humans and *E. granulosus* infection in dogs, (ii) was published in English, (iii) disclosed the number of individuals studied and the number of cases, (iv) disclosed study location, (v) was carried out and published between 1 January 1970 and 31 December 2021, (vi) identified the cause of echinococcosis in humans and dogs as the larva and adult of *E. granulosus* respectively and (vii) disclosed the method of diagnosis employed.

To ensure the quality of our data and reduce the likelihood of errors, four authors (SNK, NBA, MZ and KM) participated in the screening of articles, their selection as well as quality assessment and extraction of data independently. However, in cases of discrepancies, the four authors cross checked data simultaneously with the help of two others (MIA and AAM) and discuss issues until consensus was reached. Data extracted from each relevant article included: (i) surname of author, (ii) the year of conduct and publication of a study, (iii) the number of individuals examined by each article and the number of cases, (iv) the location where the study was conducted and (v) finally the method of diagnosis employed.

### Risk of bias within study

We examined within-study bias using the Joanna Briggs Institute (JBI) critical appraisal instrument for studies reporting prevalence data published by Munn et al. [17], which is available from https://pubmed.ncbi.nlm.nih.gov/26317388. The JBI checklist poses nine questions focusing on: (i) suitability of sample frame, (ii) suitability of the way study participants were sampled, (iii) sufficiency of sample size, (iv) exhaustiveness of the description of study subjects and settings, (v) sufficiency of data analysis of the identified sample, (vi) soundness of the methods employed for the detection of human cystic echinococcosis and canine *E. granulosus* infection, (vii) dependability of the measurement of the condition in all participants, (viii) relevance of the statistical analysis used and (ix) sufficiency of the response rate and its management. Based on these nine questions of the JBI checklist, we scored articles 0, 1 or NA for having no, yes or not applicable response to a question. With slight modifications adopted from Karshima et al. [18], we grouped articles with total score ranges of 0–3 as having high risk of bias, 4–6 as having moderate risk and 7–9 as having low risk.

### Pooling and heterogeneity analysis

Data were entered through Microsoft Excel, cleaned and subjected to statistical and meta-analysis using StataMP version 14 and Comprehensive Meta-Analysis version 3.0. We determined the prevalence of each recruited article and its 95% confidence interval (CI) by employing the online exact binomial proportion and CI calculator available from http://statpages.info/confint.html. Estimated prevalence of HCE and EGI in dogs and their respective 95% CI were evaluated using the random-effects model of meta-analysis with the assumption that the true effect sizes may differ within recruited articles since they were carried out using different methodologies and under different environmental conditions [19]. Heterogeneity was determined using Cochran’s Q-test while the degree of variation across studies was quantified by the *I*-square statistics. According to the method of Higgins et al. [20], absence of heterogeneity, low, moderate and substantive heterogeneities were represented by *I*^2^ values of 0, 25, 50 and 75% respectively.

### Publication bias and sensitivity analysis

Publication bias was assessed using funnel plot while its statistical significance was measured by Egger’s regression asymmetry test [21]. We used the non-parametric “fill and trim” linear random method described by Duval and Tweedie [22] to test for unbiased estimates. To test for sensitivity, we deleted one article at any given point before carrying out meta-analysis until analysis was done without each of the relevant articles. Any estimated prevalence (EP) value that was within the 95% CI of the overall EP, when number of articles equals N-1, affirms that the deleted study did not significantly influence the present analysis [23].

### Sub-grouping and meta-regression

We performed sub-group analysis for sub-regions of Africa (Central, Eastern, Northern, Southern and Western), methods of diagnosis (others, serology and ultrasonography) for HCE and (ELISA, microscopy and PCR) for EGI, study periods (1970–1987, 1988–2004 and 2005–2021) for HCE and (1975–1990, 1991–2006 and 2007–2021) for EGI, sample sizes of relevant articles (≤ 500, 501–1000 and > 1000) for HCE and (≤ 200, 201–400 and > 400) for EGI, gender/sex (men and women) for HCE and (females and males) for EGI, age (adult and children) and dog type (owned and stray dogs).

We also performed meta-regression to identify possible sources of heterogeneity in the analysis for HCE and compared Western Africa with other sub-regions from Africa, ultrasonography with other diagnostic methods, 2005–2021 with other study periods, sample size > 1000 with other sample sizes, men with women and finally children with adults. For EGI in dogs, we also compared Western Africa with other sub-regions from Africa, PCR with other diagnostic methods, 2007–2021 with other study periods, sample size > 400 with other sample sizes, male dogs with females and stray dogs with owned dogs.

## Results

### Human cystic echinococcosis in Africa

**Fig. 1:**
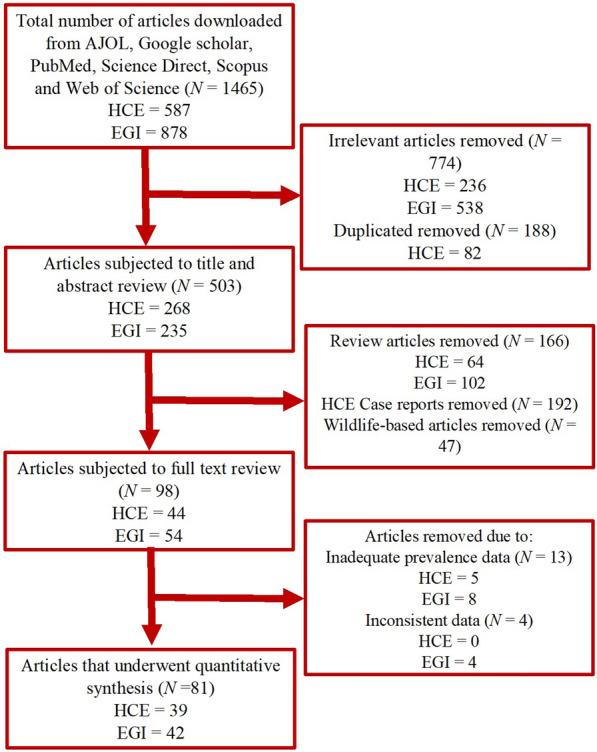
Flow diagram for the selection of eligible studies

The process for the selection of articles on HCE is presented in Fig. 1. A total of 587 articles on HCE in Africa were identified. Of these, 39 articles [12, 59–96] that certified the inclusion criteria were synthesized after the removal of 205 irrelevant, 82 duplicate, 64 review articles, 192 case reports and 5 others with inadequate prevalence data. The characteristics of the articles on HCE are presented in Table 1. The majority (53.9%) of the articles on HCE were from Northern Africa. Five articles diagnosed HCE using other methods (one autopsy, one surgery and three combinations of different methods), 16 used serology and the remaining 18 utilized ultrasonography. The 39 articles on HCE were spread across study period with 13, 10 and 16 of them respectively conducted during 1970 and 1987, 1988 and 2004 as well as 2005 and 2021. Furthermore, 13, 8 and 18 of the articles respectively reported sample sizes of ≤ 500, 501–1000 and > 1000 while 10 articles each reported HCE in adults and children. Finally, 5 articles scored 7–9 points based on the JBI critical appraisal checklist and were classified as articles with low risk of bias, while 34 of them scored 4–6 points and were classified as having moderate risk of bias.

**Table 1:** List and characteristics of studies on human cystic echinococcosis in Africa

Country	Study year	MOD	Sample size	Cases	Prev. (%)	95% CI	ROB	Study reference
C/Africa								
Gabon	2014–16	US	348	0	0.00	0.00, 1.05	MR	Lotsch et al. [59]
E/Africa								
Ethiopia	2008–12	US	25,840	27	0.10	0.07, 0.15	LR	Assefa et al. [60]
Ethiopia	1980	IHT	1342	68	3.49	2.60, 4.57	MR	Fuller and Fuller [61]
Ethiopia	2002–06	US	36,402	26	0.07	0.05, 0.10	MR	Kebede et al. [62]
Ethiopia	1989	US	990	7	6.41	5.16, 7.85	MR	Klungsoyr et al. [63]
Ethiopia, Kenya, South Sudan and Tanzania	1985–87	US	18,565	332	1.79	1.60, 1.99	MR	Macpherson et al. [64]
Kenya	1981/82	IHT	1190	85	7.14	5.74, 8.76		French and Ingera [65]
Kenya	1991	ELISA	538	88	16.36	13.33, 19.76	MR	Kenny and MacCabe [66]
Kenya	1986	USS/ELISA	3553	198	5.57	4.84, 6.38	MR	Macpherson et al. [67]
Kenya	1983–15	US	961	240	24.97	22.27, 27.84	MR	Solomon et al. [68]
Kenya	1985–12	US	10,920	418	3.83	3.48, 4.20	MR	Solomon et al. [69]
Mozambique	2011/11	MWB	601	104	17.30	14.36, 20.57	MR	Noormahomed et al. [70]
South Sudan	2012	USS	610	4	0.66	0.18, 1.67	MR	Stewart et al. [71]
Tanzania	2012	ELISA	345	39	11.30	8.16, 15.13	MR	Khan et al. [72]
Tanzania	1977–86	US	959	10	1.04	0.50, 1.91	MR	Macpherson et al. [73]
N/Africa								
Egypt	1974	LAT	755	47	6.23	4.61, 8.19	MR	Botros et al. [74]
Egypt	1997–99	MRI, US & X-ray	492,353	133	0.03	0.02, 0.03	MR	Kandeel et al. [75]
Egypt	2006	IHT	21	3	14.29	3.05, 36.34	MR	Mazyat et al. [76]
Libya	1972–79	Surgical	22,979	111	0.48	0.40, 0.58	MR	Aboundaya [77]
Libya	1979–80	ELISA	217	20	9.22	5.72, 13.88		Gebreel et al. [78]
Libya	1989	IHT	384	8	0.36	0.13, 0.79	MR	Khan et al. [79]
Libya	2008–11	ELISA	300	27	9.00	6.01, 12.82	LR	Mohamed et al. [80]
Libya	1991	US	4103	57	1.39	1.05, 1.81	MR	Shambesh et al. [81]
Libya	1996	US	485	22	4.54	2.86, 6.79	MR	Shambesh et al. [82]
Libya	1998	US	20,220	339	1.68	1.50, 1.86	MR	Shambesh et al. [83]
Morocco	2014	US	5367	102	1.90	1.55, 2.30	LR	Chebil et al. [84]
Morocco	2000/01	US	11,612	126	1.09	0.90, 1.29	MR	Macpherson et al. [85]
Sudan	2017/18	ELISA	305	20	6.56	4.05, 9.95	LR	Ahmed et al. [86]
Sudan	2002	US	300	1	0.33	0.01, 1.84	LR	Elmahdi et al. [87]
Tunisia	1980–84	ELISA	355	8	2.25	0.98, 4.39	MR	Bchir et al. [88]
Tunisia	1990	US	1434	50	1.34	0.62, 2.53	MR	Bchir et al. [89]
Tunisia	1990–17	US	7808	26	0.33	0.22, 0.49	MR	Jomaa et al. [90]
Tunisia	2004–09	Autopsy	2155	26	2.08	0.90, 4.06	MR	Khelil et al. [91]
Tunisia	2018	ELISA	374	32	8.56	5.93, 11.86	MR	M’rad et al. [92]
Tunisia	1983	US	670	9	0.57	0.01, 3.13	MR	Mlika et al. [93]
Tunisia	1985	US/ELISA	1650	6	13.12	9.41, 17.63	MR	Mlika et al. [94]
S/Africa								
South Africa	1995–10	IHT	236	26	11.02	7.32, 15.72	MR	Wahlers et al. [12]
W/Africa								
Nigeria	1977	AGDT and IHT	189,861	1	0.00	0.00, 0.00	MR	Dada [95]
Nigeria	1986	CFT	176	1	1.21	0.79, 1.76	MR	Sixl et al. [96]

*AGDT* Agar gel diffusion test, *CFT* complement fixation test, *CI* confidence interval, *C/Africa* Central Africa, *E/Africa* eastern Africa, *ELISA* enzyme-linked immunosorbent assay, *IHT* indirect hemagglutination test, *LAT* latex agglutination test, *LR* low risk, *MR* moderate risk, *MRI* magnetic resonance imaging, *MWB* multiplex western blot, *N/Africa* northern Africa, *ROB* risk of bias, *S/Africa* southern Africa, *US* ultrasonography, *W/Africa* western Africa

**Fig. 2:**
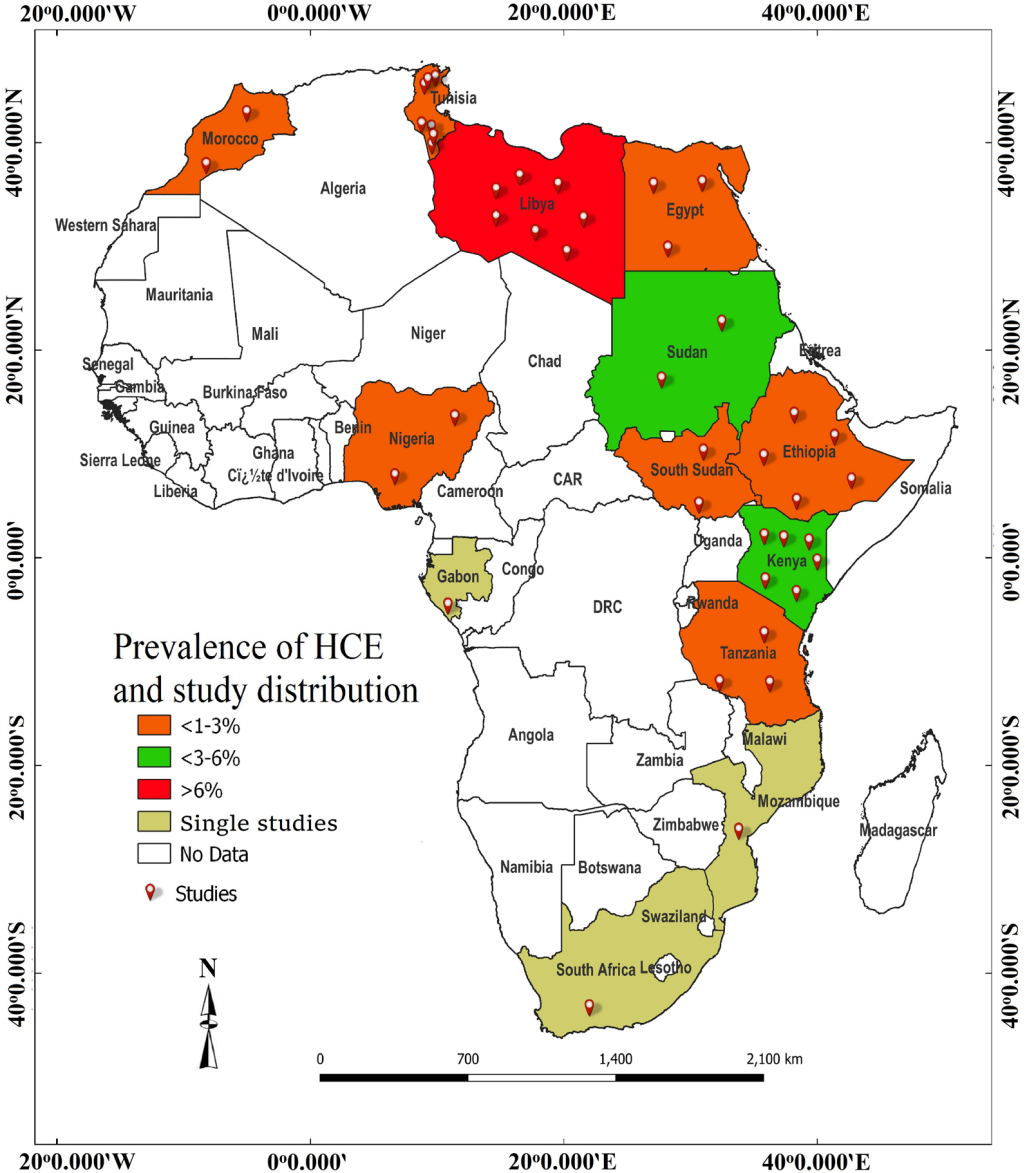
Country-based prevalence and distribution of articles on HCE

**Fig. 3:**
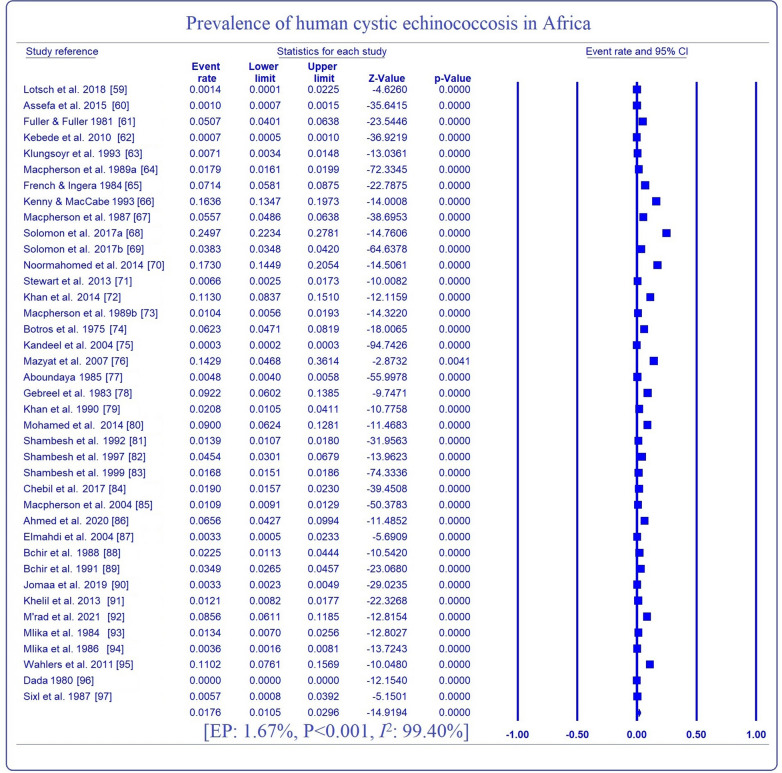
Forest plot for the prevalence of human cystic echinococcosis in Africa

Table 2 shows the estimated prevalence of HCE in Africa. The overall EP of HCE was 1.7% with a statistically significant (*P* < 0.001) sub-group range of 0.0% (95% CI 0.0, 14.1) to 11.0% (95% CI 7.6, 15.7). Highest EPs of HCE were observed in Eastern Africa (2.7%, 95% CI 1.4, 5.4), serology (5.8%, 95% CI 4.0, 8.4), the study period 2005–2021 (2.6%, 95% CI 1.2, 5.8), sample size ≤ 500 (5.4%, 95% CI 3.7, 7.9), women (3.4%, 95% CI 1.9, 6.0) and adults (6.0%, 95% CI 3.3, 10.5). Country-based prevalence of HCE ranged between 0.0 (95% CI 1.2, 1.5) in Nigeria and 9.2% (95% CI 5.7, 13.9) in Libya with the highest proportion of the articles (18.0%) reported from Libya and Tunisia (Fig. 2). Substantive heterogeneity of 99.4% was observed with a range of 0.0–99.9% even after sub-group analysis (Fig. 3).

**Table 2:** Sub-group analysis for estimated prevalence of human cystic echinococcosis in Africa

Variables	No. of studies	Estimated prevalence			(95% CI)	*P*-value	Heterogeneity			Meta-regression	
		SS	Cases	Prev. (%)			*Q*-value	*I*^2^ (%)	*Q*-*P*	*P*-value	OR (95% CI)
Regions											
C/Africa	1	348	0	0.14	0.01, 2.25	< 0.001	0.00	0.000	1.000	0.001	2.12 (− 2.62, 6.85)
E/Africa	14	102,816	1646	2.71	1.35, 5.37		2303.04	99.44	< 0.001		5.08 (2.46, 7.69)
N/Africa	21	573,847	1173	1.80	0.88, 3.63		2734.56	99.27	< 0.001		4.66 (2.09, 7.24)
S/Africa	1	236	26	11.02	7.61, 15.67		0.00	0.00	1.000		6.57 (2.71, 10.44)
W/Africa	2	190,037	2	0.02	0.00, 14.06		24.36	95.89	< 0.001		Reference
MOD											
Others	5	522,690	474	0.51	0.05, 5.04	< 0.001	2274.46	99.82	< 0.001	0.002	− 0.78 (− 2.26, 0.69)
Serology	16	197,000	577	5.80	3.98, 8.39		265.29	94.35	< 0.001		1.46 (0.44, 2.48)
US	18	147,594	1796	1.13	0.62, 2.03		2241.26	99.24	< 0.001		Reference
Study period											
1970–1987	13	242,272	896	1.55	0.84, 2.84	< 0.001	786.20	98.47	< 0.001	0.438	− 0.69 (− 1.92, 0.54)
1988–2004	10	532,419	831	1.30	0.36, 4.60		2621.83	99.66	< 0.001		− 0.71 (− 2.03, 0.60)
2005–2021	16	92,593	1120	2.64	1.18, 5.82		2145.82	99.30	< 0.001		Reference
Sample size											
≤ 500	13	3846	207	5.44	3.73, 7.89	< 0.001	73.06	83.58	< 0.001	0.001	1.81 (0.78, 2.83)
501–1000	8	6084	509	4.13	1.92, 8.65		369.19	98.10	< 0.001		1.75 (0.58, 2.92)
> 1000	18	857,354	2131	0.71	0.36, 1.41		3968.06	99.57	< 0.001		Reference
Gender											
Women	17	39,667	758	3.39	1.90, 5.97	< 0.001	896.15	98.22	< 0.001	0.523	0.29 (− 0.59, 1.16)
Men	17	33,266	428	2.54	1.34, 4.76		581.00	97.25	< 0.001		Reference
Age											
Adult	10	10,351	411	5.98	3.33, 10.51	< 0.001	260.91	96.55	< 0.001	0.045	1.12 (0.03, 2.20)
Children	10	11,962	124	1.96	0.66, 5.68		256.39	96.49	< 0.001		Reference
Overall	39	921,794	3713	1.67	1.08, 2.58		6297.68	99.40	< 0.001		

*CI* confidence interval, *C/Africa* Central Africa, *ELISA* enzyme linked immunosorbent assay, *E/Africa* eastern Africa, *I*^2^ inverse variance index, *MOD* method of diagnosis, *N/Africa* northern Africa, *OR* odds ratio, *PCR* polymerase chain reaction, *Prev* prevalence, *Q-P* Cochrane’s *P*-value, *SS* sample size, *S/Africa* southern Africa, *US* ultrasonography, *W/Africa* western Africa

**Fig. 4:**
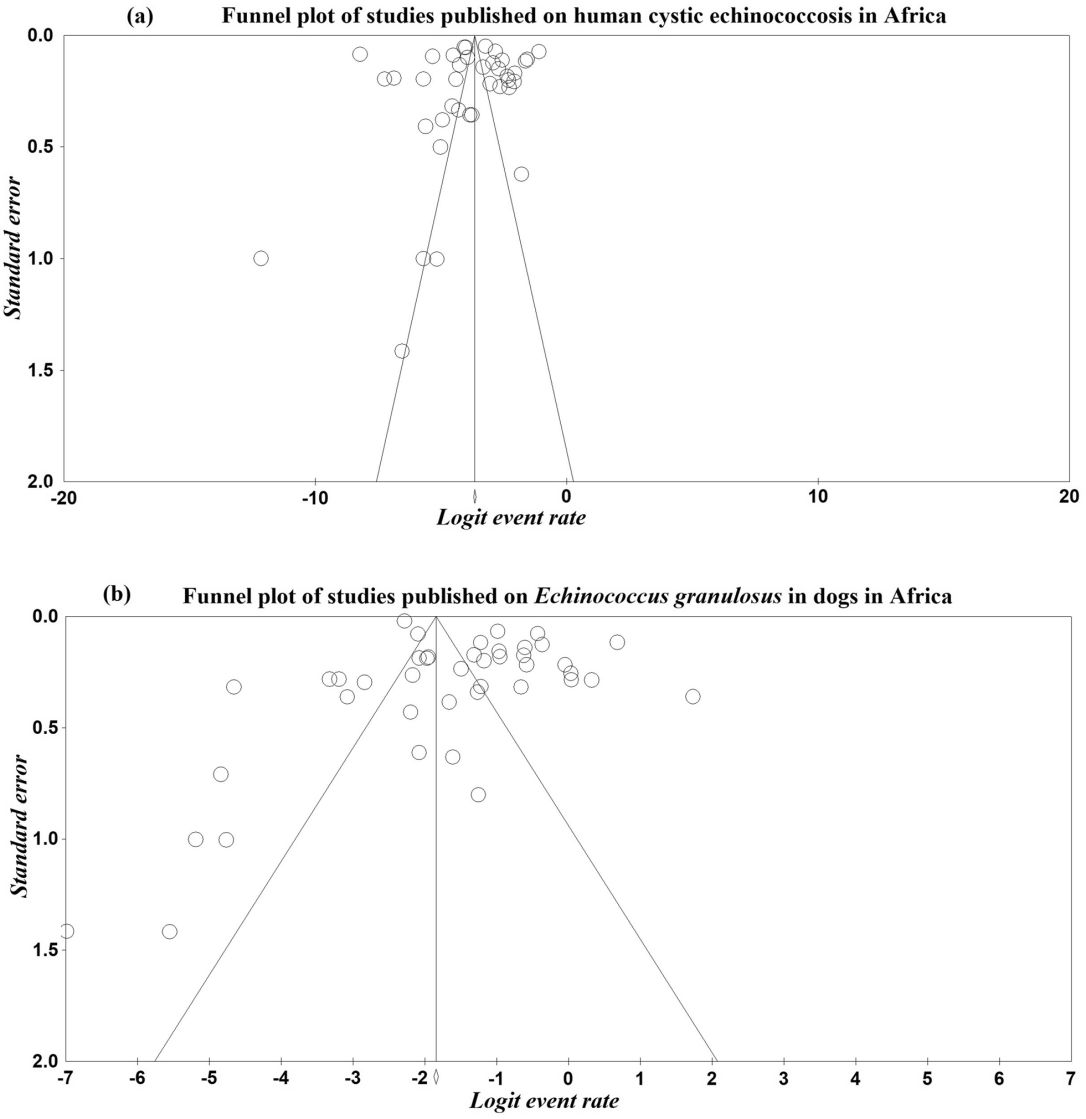
Funnel plot for studies published on **a** HCE and **b** EGI in dogs

Funnel plot for articles published on HCE (Fig. 4a) and findings from Egger regression test indicated insignificant publication bias. As shown by the results of our sensitivity analysis, no single article influenced the results of the present analysis (Additional file 1: Dataset S1). Meta-regression analysis implicated study locations, methods of diagnosis, sample sizes of individual articles and age of participants (*P* < 0.05) as possible sources of the heterogeneity associated with the analysis on HCE (Table 2).

### *Echinococcus granulosus* infection in dogs in Africa

Overall, 878 articles were identified, but following screening, a total of 538 irrelevant, 137 duplicates, 102 review articles, 47 wildlife-based articles as well as 8 articles with inadequate prevalence and 4 with inconsistent data were excluded (Fig. 1). Forty-two articles [53, 55–57, 59, 97–131] were finally synthesized. The characteristics of the 42 articles that reported EGI in dogs are presented in Table 3. A higher proportion (20/42) of the articles on EGI were from Northern Africa. Three, 12 and 27 of the articles diagnosed *E. granulosus* using ELISA, PCR and microscopy respectively. Additionally, 15, 9 and 18 of the studies were conducted between 1975 and 1990, 1991 and 2006 as well as 2007 and 2021 respectively. Furthermore, 25, 10 and 18 of the articles had sample sizes of ≤ 200, 201–400 and > 400 respectively. Finally, based on the JBI critical appraisal checklist, 38 of the articles were grouped as articles of moderate risk of bias (4–6 points) and the remaining 4 as those with low risk of bias (7–9 points) as shown in Table 3.

**Table 3:** List and characteristics of studies on *Echinococcus granulosus* infections in dogs in Africa

Country	Study year	MOD	Sample size	Cases	Prev. (%)	95% CI	ROB	Study reference
C/Africa								
Gabon	2014–16	PCR	128	0	0.00	0.00, 2.84	MR	Lotsch et al. [59]
E/Africa								
Ethiopia	2008	Microscopy	18	3	16.67	3.58, 41.42	MR	Kebede et al. [97]
Ethiopia	2010	Microscopy	44	15	34.09	20.49, 49.92	MR	Koskei et al. [98]
Ethiopia	1992	Microscopy	9	2	22.22	2.81, 60.01	MR	Mersie [99]
Kenya	2013–16	PCR–RFLP	1621	178	10.98	9.50, 12.61	MR	Mulinge et al. [57]
Kenya	2001/02	Copro-ELISA	203	56	27.59	21.56, 34.28	MR	Buishi et al. [100]
Kenya	1989	ELISA	143	50	34.97	27.19, 43.38	MR	Jenkins et al. [101]
Kenya	1979–83	Microscopy	695	274	39.42	35.77, 43.17	MR	Macpherson et al. [102]
Kenya	1992	Microscopy	156	16	10.26	5.98, 16.12	MR	Wachira et al. [103]
Uganda	2007/08	Microscopy	327	217	66.36	60.96, 71.47	LR	Inangolet et al. [104]
Uganda	2013	Copro-PCR	261	32	12.26	8.54, 16.87	LR	Oba et al. [105]
Zambia	2005/06	Multiplex-PCR	540	0	0.00	0.00, 0.68	MR	Nonaka et al. [106]
N/Africa								
Algeria	2006/07	Microscopy	120	22	18.33	11.86, 26.43	MR	Kohil et al. [107]
Egypt	2006	Microscopy	50	8	16.00	7.17, 29.11	MR	Mazyad et al. [76]
Libya	1985–88	Microscopy	92	33	35.87	26.13, 46.54	MR	Awan et al. [108]
Libya	2006	Microscopy	50	29	58.00	43.21, 71.81	MR	Ben Musa and Sadok [109]
Libya	2001/02	Copro-PCR	409	93	22.74	18.76, 27.11	LR	Buishi et al. [110]
Libya	1986	Microscopy	151	42	27.81	20.84, 35.68	MR	Gusbi [111]
Libya	1980–82	Microscopy	27	3	11.11	2.35, 29.16	MR	Packer and Ali [112]
Morocco	2016	Copro-PCR	254	104	40.94	34.84, 47.27	MR	Amarir et al. [113]
Morocco	2010–11	Microscopy	79	224	35.27	29.02, 41.91	LR	Dakkak et al. [114]
Morocco	1985	Microscopy	57	13	22.81	12.74, 35.85	MR	Pandey et al. [115]
Morocco	1987	Microscopy	61	31	50.82	37.70, 63.86	MR	Pandey et al. [116]
Sudan	2004	Copro-PCR	84	41	48.81	37.74, 59.96	MR	Omer et al. [55]
Sudan	1985	Microscopy	49	25	51.02	36.24, 65.58	MR	Saad and Magzoub [117]
Tunisia	2014	PCR	1095	298	27.21	24.60, 29.96	MR	Chaabane-Banaoues et al. [53]
Tunisia	2018	PCR	288	32	11.11	7.73, 15.32	MR	M’rad et al. [92]
Tunisia	1986	Microscopy	50	11	22.00	11.53, 35.96	MR	Bchir et al. [118]
Tunisia	2015	Microscopy	140	33	23.57	16.82, 31.48	MR	Iraqi [119]
Tunisia	1998/99	Microscopy	198	42	21.21	15.74, 27.57	MR	Lahmar et al. [120]
Tunisia	2007	Microscopy	375	13	3.47	1.86, 5.86	MR	Lahmar et al. [121]
Tunisia	2007	Copro-PCR	60	6	10.00	3.76, 20.51	MR	Lahmar et al. [122]
S/Africa								
South Africa	1978	Microscopy	1063	10	0.94	0.45, 1.72	MR	Verster [123]
W/Africa								
Mali	2010/11	Multiplex-PCR	118	1	0.85	0.02, 4.63	MR	Mauti et al. [56]
Nigeria	2012/13	ELISA	273	34	12.45	8.78, 16.97	MR	Adediran et al. [124]
Nigeria	1983	Microscopy	60	51	85.00	73.43, 92.90	MR	Arene [125]
Nigeria	2018/19	Multiplex PCR	217	12	5.53	2.89, 9.46	LR	Awosanya et al. [126]
Nigeria	1978	Microscopy	180	1	0.56	0.01, 3.06	MR	Dada et al. [127]
Nigeria	1979	Microscopy	330	13	3.94	2.11, 6.44	MR	Dada [128]
Nigeria	2019	Microscopy	26,844	2486	9.26	8.92, 9.61	MR	Karshima et al. [129]
Nigeria	1985	Microscopy	182	8	4.40	1.92, 8.48		Okolo [130]
Nigeria	1984	Microscopy	254	2	0.79	1.10, 2.82	MR	Ugochukwu and Ejimadu [131]

*CI* confidence interval, *C/Africa* Central Africa, *E/Africa* eastern Africa, *ELISA* enzyme-linked immunosorbent assay, *LR* low risk, *MR* moderate risk, *N/Africa* northern Africa, *PCR* polymerase chain reaction, *RFLP* restriction fragment length polymorphism, *ROB* risk of bias, *S/Africa* southern Africa, *W/Africa* western Africa

**Fig. 5:**
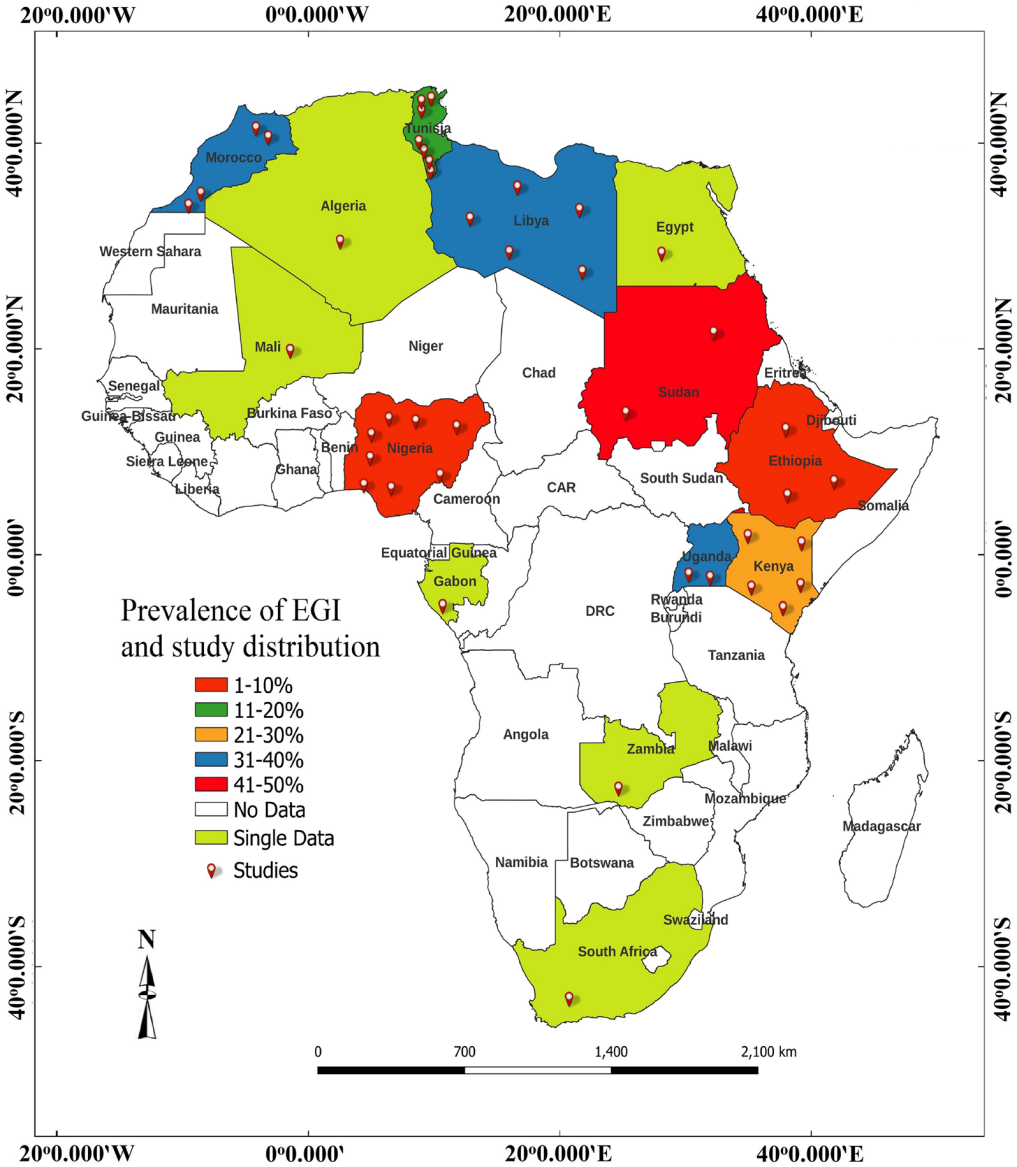
Country-based prevalence and distribution of articles on EGI

**Fig. 6:**
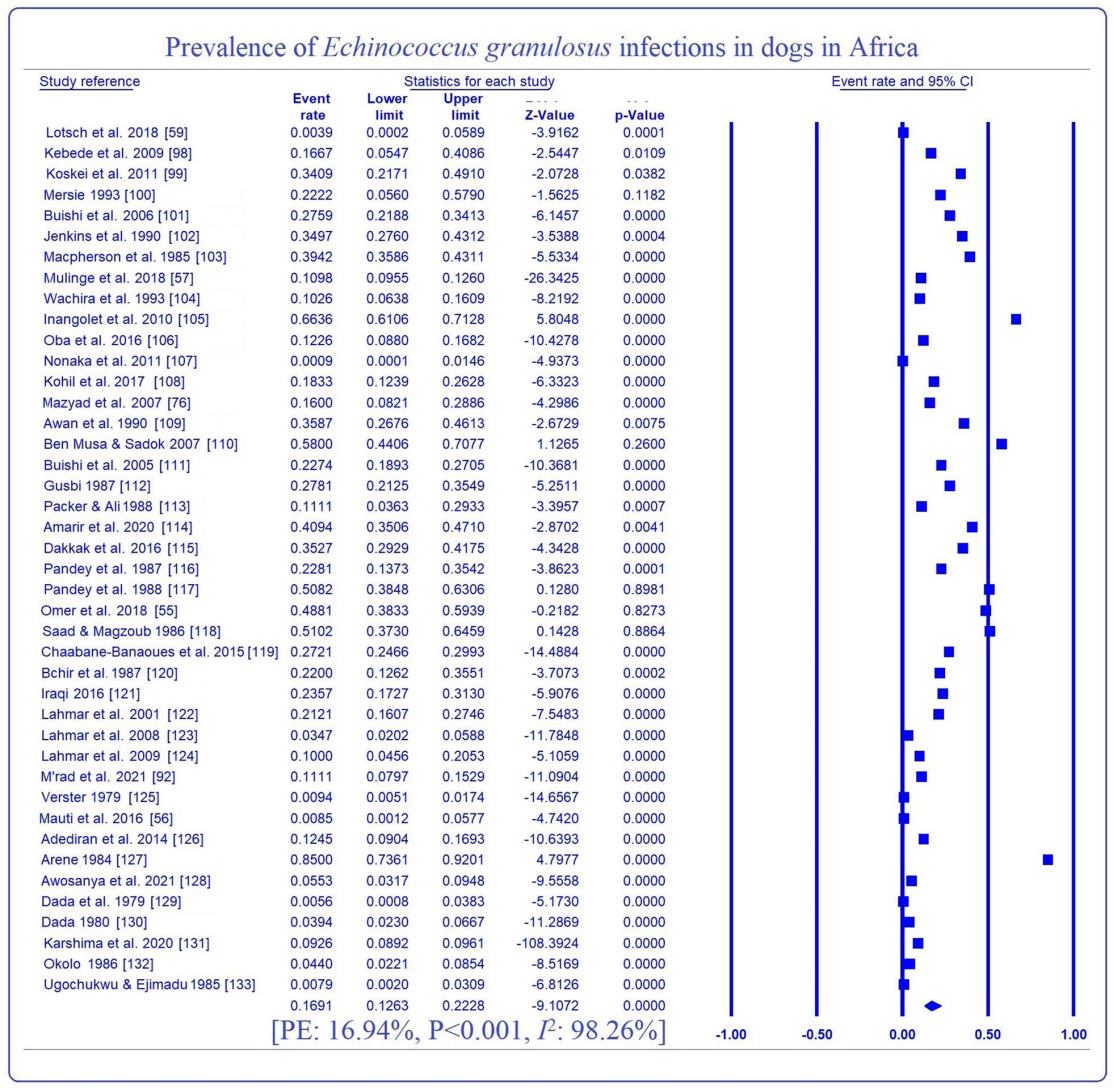
Forest plot for the prevalence of *Echinococcus granulosus* infection in dogs in Africa

EP of EGI in dogs is shown in Table 4. The overall EP was 16.9% (95% CI 12.7, 22.3) with a significant (*P* < 0.001) variation of 0.4 (95% CI 0.0, 5.9) to 35.8% (95% CI 25.4, 47.8) across sub-groups. Highest prevalences of EGI were observed in Northern Africa (25.6%, 95% CI 20.4, 31.6), ELISA detection method (23.6%, 95% CI 12.8, 39.4), study period 1991–2006 (23.4%, 95% CI 15.3, 34.0), the sample size ≤ 200 (23.5%, 95% CI 17.5, 30.8), female dogs (35.8%, 95% CI 25.4, 47.8) and stray dogs (29.7%, 95% CI 23.2, 37.0). EPs of canine EGI in relation to individual countries ranged between 2.9 (95% CI 1.9, 4.1) in Ethiopia to 49.6% (95% CI 41.2, 58.1) in Sudan, with the highest proportion of articles (19.1%) reported from Nigeria (Fig. 5). Overall, heterogeneity was 98.3% with a range of 0.0–99.4% (Table 4, Fig. 6).

**Table 4:** Sub-group analysis for estimated prevalence of *Echinococcus granulosus* infection in dogs in Africa

Variables	No. of studies	Estimated prevalence			(95% CI)	*P*-value	Heterogeneity			Meta-regression	
		SS	Cases	Prev. (%)			*Q*-value	*I*^2^ (%)	*Q*-*P*	*P*-value	OR (95% CI)
Regions											
C/Africa	1	128	0	0.39	0.02, 5.89	< 0.001	0.00	0.00	1.000	< 0.001	− 2.95 (− 6.31, 0.41)
E/Africa	11	4017	843	21.01	11.61, 35.01		519.54	98.08	< 0.001		1.25 (0.38, 2.12)
N/Africa	20	3834	958	25.62	20.44, 31.58		233.59	91.87	< 0.001		1.52 (0.73, 2.30)
S/Africa	1	1063	10	0.94	0.51, 1.74		0.00	0.00	1.000		− 2.06 (− 4.05, − 0.06)
W/Africa	9	28,458	2608	6.53	3.12, 13.15		172.88	95.37	< 0.001		Reference
MOD											
ELISA	3	619	140	23.58	12.76, 39.42	< 0.001	29.55	93.23	< 0.001	0.298	0.91 (− 0.61, 2.42)
Microscopy	27	31,806	3482	18.44	11.86, 27.53		1785.75	98.54	< 0.001		0.61 (− 0.24, 1.46)
PCR	12	5075	797	13.18	8.36, 20.17		303.58	96.38	< 0.001		Reference
Study period											
1975–1990	15	3394	567	16.61	9.24, 28.06	< 0.001	412.06	96.60	< 0.001	< 0.001	− 2.31 (− 3.55, − 1.08)
1991–2006	9	1699	287	23.35	15.26, 34.00		86.84	90.79	< 0.001		0.70 (0.08, 1.33)
2007–2021	18	32,407	3565	15.07	9.79, 22.49		1275.55	98.67	< 0.001		Reference
Sample size											
≤ 200	25	2451	565	23.48	17.49, 30.76	< 0.001	257.59	90.68	< 0.001	0.137	0.41 (− 1.10, 1.92)
201–400	10	2782	515	11.72	5.17, 24.45		512.24	98.24	< 0.001		0.74 (0.01, 1.47)
> 400	17	32,267	3339	10.44	5.30, 19.53		932.95	99.36	< 0.001		
Sex											
Female	4	868	350	35.79	25.35, 47.77	0.224	40.53	90.13	< 0.001	0.855	0.07 (− 0.66, 0.79)
Male	4	910	341	33.92	23.23, 46.56		49.44	91.91	< 0.001		Reference
Dog type											
Owned	15	3351	506	15.92	10.89, 22.71	< 0.001	229.88	93.91	< 0.001	0.006	− 0.77 (− 1.32, − 0.22)
Stray	21	1672	491	29.65	23.22, 37.01		150.59	86.72	< 0.001		Reference
Overall	42	37,500	4419	16.94	12.65, 22.32		2355.16	98.26	< 0.001		

*CI* confidence interval, *C/Africa* Central Africa, *ELISA* enzyme-linked immunosorbent assay, *E/Africa* eastern Africa, *I*^2^ inverse variance index, *MOD* method of diagnosis, *N/Africa* northern Africa, *OR* odds ratio, *PCR* polymerase chain reaction, *Prev* prevalence, *Q-P*: Cochrane’s *P*-value, *SS* sample size, *S/Africa* southern Africa, *W/Africa* western Africa

Funnel plot (Fig. 4b) and results of Egger regression test revealed insignificant publication bias across articles published on EGI in Africa. Sensitivity analysis revealed insignificant influence of individual studies on the present analysis (Additional file 2: Dataset S2). Meta-regression showed that study location (*P* < 0.001, df: 4, *Q*: 30.97), study period (*P* < 0.001, df: 2, *Q*: 27.17) and dog type (*P*: 0.006, df: 1, *Q*: 7.62) might be the possible sources of the heterogeneity in our analysis on EGI (Table 4).

## Discussion

### Human cystic echinococcosis

Human cystic echinococcosis is endemic in many African countries; however, the actual data on its current incidence, prevalence and burden are lacking. Though a number of surveillance reports are documented from different African countries, no harmonized report is available. This meta-analysis provided information on the prevalence and distribution of the disease across Africa to enable policy makers to take informed decisions towards cost-effective disease control.

The present study revealed an Africa-wide prevalence of 1.7%, which is lower than the ranges reported in Asia (2.2–6.0%) [24–27] and Latin America (4.7–7.1%) [28–30]. On the other hand, the present finding is higher than reports from European countries like Italy (0.2%) [31] and Slovakia (0.6%) [32]. These variations might be due to location-based differences in the numbers of free-roaming dogs and ownership of dogs, which are the definitive hosts that are capable of contaminating the environment. Slaughtering livestock at home, feeding raw viscera to dogs as well as environmental and climatic factors including temperature, rainfall and humidity that affect the environmental survival of eggs might also be responsible.

Our analysis revealed regional variation in the prevalence of HCE with the highest in Eastern Africa, and Northern Africa in second place. This finding affirms existing reports that the disease is highly endemic among the nomadic pastoralist of Eastern Africa [33–35]. In line with the present finding, regional variations in the prevalence of HCE were also reported in other continents such as Asia [27, 36, 37] and Latin America [28–30].

Three methods, serology, ultrasonography and “others” (autopsy, surgery and test combinations), were employed by the 39 studies for the detection of HCE. Of these methods, serology revealed the highest prevalence of the disease probably due to factors including the high sensitivity, specificity and diagnostic accuracy of these techniques, their ability to detect small-sized cyst that might be missed by ultrasonography [38] as well as the prolonged persistence of antibodies against hydatid cyst even after chemotherapy and/or surgical removal of the cysts [39]. In addition to the prevalence detected by the analyzed articles using ultrasonography, which is the gold test for the diagnosis of cystic echinococcosis in humans, it is able to identify the location, number and size of cysts [38].

Our analysis revealed a 1.0% (1.6–2.6%) increase in the prevalence of HCE during the 51 years (1970–2021) under review. Detail analysis showed an initial decline of 0.3% between 1988 and 2004, which was followed by an increase of 1.3% during 2005 and 2021. These fluctuations indicate possible inconsistencies in the control measures against the disease in Africa. The study also revealed decrease in the prevalence of HCE as sample sizes were increasing. This could indicate potential sampling biases in the articles with smaller samples, thus creating potential uncertainty in the estimated prevalence.

Prevalence of HCE in women was significantly higher than that observed among men. This may be attributable to hormonal imbalances associated with pregnancy and lactation in women which usually interfere with immunity. The finding of higher prevalence in women concurs reports from Argentina [29] and Iran [24]. Our finding however contradicts the reports of Andrabi et al. [25] from India and Acosta-Jamett et al. [40] from Chile who reported higher prevalence of HCE in men compared to women. The higher prevalence observed in adults (≥ 18 years) compared to children (≤ 17 years) is in line with the report of Uchiumi et al. [30] from Argentina and could be attributable to the chronic nature of the disease.

### *Echinococcus granulosus* infection in dogs

The second part of this study investigated the current status of *E. granulosus* in dogs which are the definitive host of the pathogen. Forty-two studies that met the inclusion criteria were harmonized to determine the prevalence and distribution of *E. granulosus*, whose metacestode is associated with the zoonosis; HCE and serious economic losses in the meat industry.

The 16.9% Africa-wide prevalence of EGI observed in dogs falls within the ranges documented elsewhere. For instance, studies from Asia reported a range of 7.3 to 48.0% [41–44], those from Latin America reported a range of 9.3 to 42.3% [45–48], and in Australia, a range of 3.16 to 50.7% was also reported [49–51] affirming reports that *E. granulosus* is cosmopolitan in distribution. The infection in these dogs may pose the risk of environmental contamination which may in turn result in the infection of intermediate hosts like domestic ruminants thereby maintaining the disease.

Regional variations in the prevalence of *E. granulosus* were observed across Africa with the highest in Northern Africa and Eastern Africa in second place, thus showing an almost similar disease pattern with HCE. These variations may be attributable to factors including differences in the level of incursion of wild dogs into peri-urban environments [52], densities of intermediate hosts as well as both the climatic and environmental influences on egg development and survival [53]. Other factors may include regional variations in the number of straying dogs, accessibility of dogs to organs of livestock infected with *E. granulosus* cysts and illegal or uninspected home slaughter [10].

Serology, particularly ELISA, revealed the highest prevalence of *E. granulosus* probably due to the high sensitivity and specificity of this method as well as its inability to distinguish between active and convalescent infections [54]. Interestingly, serology also revealed highest prevalence in HCE. Of the three techniques (ELISA, microscopy and PCR) employed in the detection of *E. granulosus*, PCR revealed the least prevalence of 14.5%. However, in addition to the prevalence reported by these PCR techniques they were able to identify genotypes such as *E. granulosus* (s.s.) (G1–G3) in Sudan, *E. canadensis* (G6–G9) in Sudan [55] and Mali [56], *E. ortleppi* (G5) and *E. felidis* (lion strain) in Kenya [57].

We observed a significant decrease in the prevalence of *E. granulosus* with an overall decline of 0.7% between 1975 and 2021. However, sub-group analysis by year of study revealed an initial increase of 6.7% between 1991 and 2006, which was followed by 7.4% decline during 2007–2021. This is suggestive of possible challenges with existing control programs against the pathogen in Africa. Similar to the finding in HCE, prevalence of *E. granulosus* significantly decreased with increasing sample size.

Hormonal imbalances associated with pregnancy and lactation, which usually compromise immunity in females, may be responsible for the higher prevalence of *E. granulosus* observed in females compared to male dogs. This finding concurs with the report of Liu et al. [58] from China. It however contradicts other reports from Iran [41, 43], Chile [47] and Australia [51]. Our study also revealed a significantly higher prevalence of *E. granulosus* in stray than owned dogs. A number of factors including easy access of stray dogs to offal of slaughtered ruminants in abattoirs and possible capture and consumption of wild ruminants during hunting may be responsible for the higher prevalence among this group.

### Meta-analysis

The heterogeneity between studies on HCE was attributed to study locations, diagnostic methods and sample sizes of the analyzed studies as well as the ages of the participants. However, that between studies on *E. granulosus* was due to study location, study periods and dog type. The present study revealed insignificant publication bias for both studies on HCE and *E. granulosus* by funnel plot and Egger regression test. Sensitivity test also showed insignificant single study influence on our analysis affirming the credibility and reliability of the present study.

### Study limitations

The study had limitations such as uneven distribution of studies on both HCE and *E. granulosus* across the continent and sub-regions. Over 41% of the studies analyzed for HCE used serology, which is incapable of differentiating active from convalescent infections. Majority of the studies on *E. granulosus* used microscopy and were unable to identify the genotypes involved in infections. In addition, only studies published in English were included in the present analysis resulting in language bias.

## Conclusion

In this study, we provided information on the prevalence and distribution of HCE and EGI in dogs in Africa. Prevalence of HCE was generally low with the highest sub-regional prevalence in Eastern Africa. Prevalence of EGI in dogs was moderately high with the highest in Northern Africa. Gender and age influenced the prevalence of HCE. Straying of dogs also influenced dog infection with *E. granulosus*. We recommend a holistic control approach that targets humans, livestock, dogs and the environment, which all play roles in disease epidemiology. This approach should involve strategic use of anthelminthics in animals, standardized veterinary meat inspection in abattoirs, control of stray dogs to reduce environmental contamination and proper environmental sanitation. Mass screening of humans in highly endemic regions will also encourage early detection and treatment.

## Supplementary Information

**Additional file 1: Dataset S1.** Sensitivity analysis for the estimated prevalence of HCE.

**Additional file 2: Dataset S2.** Sensitivity analysis for the estimated prevalence of EGI in dogs.

## Data Availability

The data supporting the conclusion of this article are all included within the article and in Additional files 1 and 2.

## Abbreviations

AJOL: African Journals OnLine
CI: Confidence interval
EGI: *Echinococcus granulosus* infection
ELISA: Enzyme-linked immunosorbent assay
EP: Estimated prevalence
HCE: Human cystic echinococcosis
I^2^: Inverse variance index
JBI: Joanna Briggs Institute
NA: Not applicable
PCR: Polymerase chain reaction
PRISMA: Preferred Reporting Items for Systematic Reviews and Meta-Analyses

## References

[1] Nakao M, Lavikainen A, Yanagida T, Ito A (2013). Phylogenetic systematics of the genus *Echinococcus* (Cestoda: Taeniidae). Int J Parasitol.

[2] Romig T, Ebi D, Wassermann M (2015). Taxonomy and molecular epidemiology of *E. granulosus* sensu lato. Vet Parasitol.

[3] Budke C, Deplazes P, Torgerson PR (2006). Global socioeconomic impact of cystic echinococcosis. Emerg Infect Dis.

[4] Alvares Rojas CA, Romig T, Lightowlers MW (2014). *Echinococcus granulosus* sensu lato genotypes infecting humans—review of current knowledge. Int J Parasitol.

[5] World Health Organisation. The control of neglected zoonotic diseases: Community based interventions for NZDs prevention and control. Report of the third conference; 23–24 November 2010. Geneva: WHO Headquarters; 2011. http://whqlibdoc.who.int/publications/2011/9789241502528_eng.pdf. Accessed 14 Mar 2014.

[6] Craig PS, Budke CM, Schantz PM, Li T, Qiu J, Yang Y (2007). Human echinococcosis: a neglected disease?. Trop Med Health.

[7] Moro P, Schantz PM (2008). Echinococcosis: a review. Intern J Infect Dis.

[8] Thompson RC (2008). The taxonomy, phylogeny and transmission of *Echinococcus*. Exp Parasitol.

[9] World Health Organization. WHO estimates of the global burden of foodborne diseases: foodborne disease burden epidemiology reference group, 2007–2015. World Health Organization; 2015. https://apps.who.int/iris/handle/10665/199350. Accessed 18 Sept 2020.

[10] Eckert J, Deplazes P (2004). Biological, epidemiological, and clinical aspects of echinococcosis, a zoonosis of increasing concern. Clin Microbiol Rev.

[11] Torgerson PR, Macpherson CNI, Vuitton DA, Palmer SR, Soulsby L, Torgerson PR, Brown DWG (2011). Cystic echinococcosis. Oxford textbook of zoonoses: biology, clinical practice, and public health control.

[12] Wahlers K, Menezes CN, Wong M, Mogoye B, Frean J, Romig T (2011). Human cystic echinococcosis in South Africa. Acta Trop.

[13] Moro P, Schantz PM (2006). Cystic echinococcosis in the Americas. Parasitol Int.

[14] Sadjjadi SM, Mikaeili F, Karamian M, Maraghi S, Sadjjadi FS, Shariat-Torbaghan S (2013). Evidence that the *Echinococcus granulosus* G6 genotype has an affinity for the brain in humans. Int J Parasitol.

[15] Ammann RW, Eckert J (1996). Cestodes: *Echinococcus*. Gastroenterol Clin N Am.

[16] Moher D, Liberati A, Tetzlaff J, Altman DG, The PRISMA Group (2009). Preferred reporting items for systematic reviews and meta-analyses: the PRISMA statement. PLoS Med.

[17] Munn Z, Moola S, Lisy K, Riitano D, Tufanaru C (2015). Methodological guidance for systematic reviews of observational epidemiological studies reporting prevalence and cumulative incidence data. Int J Evid Based Healthc.

[18] Karshima SN, Ahmed MI, Kogi CA, Iliya PS (2022). *Anaplasma phagocytophilum* infection rates in questing and host-attached ticks: a global systematic review and meta-analysis. Acta Trop.

[19] Hedges LV, Vevea JL (1998). Fixed- and random-effects models in meta-analysis. Psychol Methods.

[20] Higgins JP, Thompson SG, Deeks JJ, Altman DG (2003). Measuring inconsistency in meta-analyses. BMJ.

[21] Egger M, Davey SG, Schneider M, Minder C (1997). Bias in meta-analysis detected by a simple, graphical test. BMJ.

[22] Duval S, Tweedie R (2000). Trim and fill: a simple funnel-plot-based method of testing and adjusting for publication bias in meta-analysis. Biometrics.

[23] Gao L, Zhang L, Jin Q (2009). Meta-analysis: prevalence of HIV infection and syphilis among MSM in China. Sex Transm Infect.

[24] Shafiei R, Teshnizi SH, Kalantar K, Gholami M, Mirzaee G, Mirzaee F (2016). The seroprevalence of human cystic echinococcosis in Iran: a systematic review and meta-analysis study. J Parasitol Res.

[25] Andrabi A, Tak H, Lone BA, Para BA (2020). Seroprevalence of human cystic echinococcosis in South Kashmir, India. Parasite Epidemiol Control.

[26] Dorjsuren T, Ganzorig S, Dagvasumberel M, Tsend-Ayush A, Ganbold C, Ganbat M (2020). Prevalence and risk factors associated with human cystic echinococcosis in rural areas, Mongolia. PLoS ONE.

[27] Yan S, Wang D, Zhang J, Mo X, Feng Y, Duan L (2021). Epidemiological survey of human echinococcosis in east Gansu, China. Sci Rep.

[28] Cohen H, Paolillo E, Bonifacino R, Botta B, Parada L, Cabrera P (1998). Human cystic echinococcosis in a Uruguayan community: a sonographic, serologic, and epidemiologic study. Am J Trop Med Hyg.

[29] Bingham GM, Budke CM, Larrieu E, Del Carpio M, Mujica G, Slater MR (2014). A community-based study to examine the epidemiology of human cystic echinococcosis in Rio Negro Province, Argentina. Acta Trop.

[30] Uchiumi L, Mujica G, Araya D, Salvitti JC, Sobrino M, Moguillansky S (2021). Prevalence of human cystic echinococcosis in the towns of Ñorquinco and Ramos Mexia in Rio Negro Province, Argentina, and direct risk factors for infection. Parasites Vectors.

[31] Manciulli T, Serraino R, D'Alessandro GL, Cattaneo L, Mariconti M, Vola A (2020). Evidence of low prevalence of cystic echinococcosis in the Catanzaro Province, Calabria Region, Italy. Am J Trop Med Hyg.

[32] Antolová D, Halánová M, Janičko M, Jarčuška P, Reiterová K, Jarošová J (2018). A community-based study to estimate the seroprevalence of trichinellosis and echinococcosis in the Roma and Non-Roma population of Slovakia. Int J Environ Res Public Health.

[33] Romig T, Omer RA, Zeyhle E, Huttner M, Dinkel A, Siefert L (2011). Echinococcosis in sub-Saharan Africa: emerging complexity. Vet Parasitol.

[34] Romig T, Deplazes P, Jenkins D, Giraudoux P, Massolo A, Craig PS (2017). Ecology and life cycle patterns of *Echinococcus* species. Adv Parasitol.

[35] Wahlers K, Menezes CN, Wong ML, Zeyhle E, Ahmed ME, Ocaido M (2012). Cystic echinococcosis in sub-Saharan Africa. Lancet Infect Dis.

[36] Yuan R, Wu H, Zeng H, Liu P, Xu Q, Gao L (2017). Prevalence of and risk factors for cystic echinococcosis among herding families in five provinces in western China: a cross-sectional study. Oncotarget.

[37] Khalkhali HR, Foroutan M, Khademvatan S, Majidiani H, Aryamand S, Khezri P (2018). Prevalence of cystic echinococcosis in Iran: a systematic review and meta-analysis. J Helminthol.

[38] Gao CH, Wang JY, Shi F, Steverding D, Wang X, Yang Y (2018). Field evaluation of an immunochromatographic test for diagnosis of cystic and alveolar echinococcosis. Parasites Vectors.

[39] Barnes TS, Deplazes P, Gottstein B, Jenkins DJ, Mathis A, Siles-Lucas M (2012). Challenges for diagnosis and control of cystic hydatid disease. Acta Trop.

[40] Acosta-Jamett G, Hernández FA, Castro N, Tamarozzi F, Uchiumi L, Salvitti JC (2022). Prevalence rate and risk factors of human cystic echinococcosis: a cross-sectional, community-based, abdominal ultrasound study in rural and urban north-central Chile. PLoS Negl Trop Dis.

[41] Mehrabani D, Oryan A, Sadjjadi SM (1999). Prevalence of *Echinococcus granulosus* infection in stray dogs and herbivores in Shiraz. Iran Vet Parasitol.

[42] Eslami A, Hosseini SH (1998). *Echinococcus granulosus* infection of farm dogs of Iran. Parasitol Res.

[43] Maleky F, Moradkhan M (2000). Echinococcosis in the stray dogs of Tehran. Iran Ann Trop Med Parasitol.

[44] Gong Q-L, Ge G-Y, Wang Q, Tian T, Liu F, Diao N-C (2021). Meta-analysis of the prevalence of *Echinococcus* in dogs in China from 2010 to 2019. PLoS Negl Trop Dis.

[45] Larrieu E, Costa MT, Cantoni G, Labanchi JL, Bigatti R, Aquino A (2000). Rate of infection and of reinfection by *Echinococcus granulosus* in rural dogs of the province of Rio Negro, Argentina. Vet Parasitol.

[46] Hoffmann AN, Malgor R, Rue ML (2001). Prevalence of *Echinococcus granulosus* (Batsch, 1786) in urban stray dogs from Dom Pedrito in the State of Rio Grande do Sul, Brazil. Cienc Rural.

[47] Acosta-Jamett G, Weitzel T, Boufana B, Adones C, Bahamonde A, Abarca K (2014). Prevalence and risk factors for echinococcal infection in a rural area of northern Chile: a household-based cross-sectional study. PLoS Negl Trop Dis.

[48] Flores V, Viozzi G, Garibotti G, Zacharias D, Debiaggi MF, Kabaradjian S (2017). Echinococcosis and other parasitic infections in domestic dogs from urban areas of an Argentinean Patagonian city. Medicina.

[49] Jenkins DJ, McKinlay A, Duolong HE, Bradshaw H, Craig PS (2006). Detection of *Echinococcus granulosus* coproantigens in faeces from naturally infected rural domestic dogs in south eastern Australia. Aust Vet J.

[50] Jenkins DJ, Lievaart JJ, Boufana B, Lett WS, Bradshaw H, Armua-Fernandez MT (2014). *Echinococcus granulosus* and other intestinal helminths: current status of prevalence and management in rural dogs of eastern Australia. Aust Vet J.

[51] Harriott L, Gentle M, Traub R, Cobbold R, Soares MR (2019). Geographical distribution and risk factors for *Echinococcus granulosus* infection in peri-urban wild dog populations. Int J Parasitol Parasites Wildl.

[52] McNeill AT, Leung LK, Goullet MS, Gentle MN, Allen BL (2016). Dingoes at the doorstep: Home range sizes and activity patterns of dingoes and other wild dogs around urban areas of North-Eastern Australia. Animals (Basel).

[53] Chaâbane-Banaoues R, Oudni-M'rad M, Cabaret J, M'rad S, Mezhoud H, Babba H (2015). Infection of dogs with *Echinococcus granulosus*: causes and consequences in an hyperendemic area. Parasites Vectors.

[54] Morel N, Lassabe G, Elola S, Bondad M, Herrera S, Marí C (2013). A monoclonal antibody-based copro-ELISA kit for canine echinococcosis to support the PAHO effort for hydatid disease control in South America. PLoS Negl Trop Dis.

[55] Omer RA, Daugschies A, Gawlowska S, Elnahas A, Kern P, Bashir S (2018). First detection of *Echinococcus granulosus* sensu stricto (G1) in dogs in central Sudan. Parasitol Res.

[56] Mauti S, Traoré A, Crump L, Zinsstag J, Grimm F (2016). First report of *Echinococcus granulosus* (genotype G6) in a dog in Bamako, Mali. Vet Parasitol.

[57] Mulinge E, Magambo J, Odongo D, Njenga S, Zeyhle E, Mbae C (2018). Molecular characterization of *Echinococcus* species in dogs from four regions of Kenya. Vet Parasitol.

[58] Liu CN, Xu YY, Cadavid-Restrepo AM, Lou ZZ, Yan HB, Li L (2018). Estimating the prevalence of *Echinococcus* in domestic dogs in highly endemic for echinococcosis. Infect Dis Pov.

[59] Lötsch F, Mombo-Ngoma G, Mischlinger J, Groger M, Veletzky L, Adegnika AA (2018). Preliminary evidence for the absence of cystic echinococcosis in Gabon: a cross-sectional pilot survey in humans and definitive hosts. Am J Trop Med Hyg.

[60] Assefa H, Mulate B, Nazir S, Alemayehu A (2015). Cystic echinococcosis amongst small ruminants and humans in central Ethiopia. Onderstepoort J Vet Res.

[61] Fuller GK, Fuller DC (1981). Hydatid disease in Ethiopia: clinical survey with some immunodiagnostic test results. Am J Trop Med Hyg.

[62] Kebede N, Mitiku A, Tilahun G (2010). Retrospective survey of human hydatidosis in Bahir Dar, north-western Ethiopia. East Mediterr Health J.

[63] Klungsøyr P, Courtright P, Hendrikson TH (1993). Hydatid disease in the Hamar of Ethiopia: a public health problem for women. Trans R Soc Trop Med Hyg.

[64] Macpherson CNL, Craig PS, Romig T, Zeyhle E, Watsghinger H (1989). Observations on human echinococcosis (hydatidosis) and evaluation of transmission factors in the Maasai of northern Tanzania. Ann Trop Med Parasitol.

[65] French CM, Ingera WE (1984). (1984) Hydatid disease in the Turkana District of Kenya. Ann Trop Med Parasitol.

[66] Kenny JV, Maccabe RJ (1993). Sero-epidemiology of hydatid disease in the non-intervention area of north-east Turkana. Ann Trop Med Parasitol.

[67] MacPherson CN, Romig T, Zeyhle E, Rees PH, Were JB (1987). Portable ultrasound scanner versus serology in screening for hydatid cysts in a nomadic population. Lancet.

[68] Solomon N, Zeyhle E, Carter J, Wachira J, Mengiste A, Romig T (2017). Cystic echinococcosis in Turkana, Kenya: the role of cross-sectional screening surveys in assessing the prevalence of human infection. Am J Trop Med Hyg.

[69] Solomon N, Zeyhle E, Subramanian K, Fields PJ, Romig T, Kern P (2018). Cystic echinococcosis in Turkana, Kenya: 30 years of imaging in an endemic region. Acta Trop.

[70] Noormahomed EV, Nhacupe N, Mascaró-Lazcano C, Mauaie MN, Buene T, Funzamo CA (2014). A cross-sectional serological study of cysticercosis, schistosomiasis, toxocariasis and echinococcosis in HIV-1 infected people in Beira, Mozambique. PLoS Negl Trop Dis.

[71] Stewart BT, Jacob J, Finn T, Lado M, Napoleon R, Brooker S (2013). Cystic echinococcosis in Mundari tribe-members of South Sudan. Pathog Glob Health.

[72] Khan MB, Sonaimuthu P, Lau YL, Al-Mekhlafi HM, Mahmud R, Kavana N (2014). High seroprevalence of echinococossis, schistosomiasis and toxoplasmosis among the populations in Babati and Monduli districts, Tanzania. Parasites Vectors.

[73] Macpherson CNL, Spoerry A, Zeyhle E, Romig T, Gorfe M (1989). Pastoralists and hydatid disease: an ultrasound scanning prevalence survey in East Africa. Trans R Soc Trop Med Hyg.

[74] Botros BA, Moch RW, Barsoum IS, Mahmoud AH, Fahmi S, El-Leil MS (1975). Echinococcosis in Egypt: IV. Serology on patients with chest problems. J Trop Med Hyg.

[75] Kandeel A, Ahmed ES, Helmy H, El Setouhy M, Craig PS, Ramzy RM (2004). A retrospective hospital study of human cystic echinococcosis in Egypt. East Mediterr Health J.

[76] Mazyad SA, Mahmoud LH, Hegazy MM (2007). *Echinococcus granulosus* in stray dogs and Echino-IHAT in the hunters in Cairo, Egypt. J Egypt Soc Parasitol.

[77] Aboudaya MA (1985). Prevalence of human hydatidosis in Tripoli region of Libya. Int J Zoonotic.

[78] Gebreel AO, Gilles HM, Prescott JE (1983). Studies on the sero-epidemiology of endemic diseases in Libya. Ann Trop Med Parasitol.

[79] Khan AH, Elsageyer MM, Kidwai SA (1990). Sero-epidemiological study of human hydatid disease in Libya using finger prick blood samples. Ann Trop Med Parasitol.

[80] Mohamed RM, Abdel-Hafeez EH, Belal US, Norose K, Aosai F (2014). Human cystic echinococcosis in the Nalut district of Western Libya: a clinico-epidemiological study. Trop Med Health.

[81] Shambesh MKL, Macpherson CN, Beesley WN, Gusbi A, Elsonosi T (1992). Prevalence of human hydatid disease in northwestern Libya: a cross-sectional ultrasound study. Ann Trop Med Parasitol.

[82] Shambesh MK, Craig PS, Ibrahem MM, Gusbi AM, Echtuish EF (1997). A high prevalence of cystic hydatid disease in North Africa. Ann Trop Med Parasitol.

[83] Shambesh MA, Craig PS, Macpherson CN, Rogan MT, Gusbi AM, Echtuish EF (1999). An extensive ultrasound and serologic study to investigate the prevalence of human cystic echinococcosis in northern Libya. Am J Trop Med Hyg.

[84] Chebli H, Laamrani EL, Idrissi A, Benazzouz M, Lmimouni BE, Nhammi H (2017). Human cystic echinococcosis in Morocco: ultrasound screening in the Mid Atlas through an Italian-Moroccan partnership. PLoS Negl Trop Dis.

[85] Macpherson CN, Kachani M, Lyagoubi M, Berrada M, Shepherd M, Fields PF (2004). Cystic echinococcosis in the Berber of the Mid Atlas mountains, Morocco: new insights into the natural history of the disease in humans. Ann Trop Med Parasitol.

[86] Ahmed ME, Abdalla SS, Adam IA, Grobusch MP, Aradaib IE (2021). Prevalence of cystic echinococcosis and associated risk factors among humans in Khartoum State, Central Sudan. Intern Health.

[87] Elmahdi IE, Ali QM, Magzoub MM, Ibrahim AM, Saad MB, Romig T (2004). Cystic echinococcosis of livestock and humans in central Sudan. Ann Trop Med Parasitol.

[88] Bchir A, Hamdi A, Jemni L, Dazza MC, Allegue M, Braham MS (1988). Serological screening for hydatidosis in households of surgical cases in central Tunisia. Ann Trop Med Parasitol.

[89] Bchir A, Larouze B, Soltani M, Hamdi A, Bouhaouala B, Ducic S (1991). Echotomographic and serological population-based study of hydatidosis in central Tunisia. Acta Trop.

[90] Jomaa SB, Salem NJ, Hmila I, Saadi S, Aissaoui A, Belhadj M (2019). Sudden death and hydatid cyst: a medicolegal study. Legal Med.

[91] Khelil MB, Allouche M, Banasr A, Gloulou F, Benzarti A, Zhioua M (2013). Sudden death due to hydatid disease: a six-year study in the Northern Part of Tunisia. J Forensic Sci.

[92] M’rad S, Chaâbane-Banaoues R, Ghrab M, Babba H, Oudni-M’rad M (2021). Human and animal cystic echinococcosis in Tataouine governorate: hypoendemic area in a hyperendemic country, myth or reality?. Parasites Vectors.

[93] Mlika N, Larouze B, Dridi M, Yang R, Gharbi S, Jemmali M (1984). Serologic survey of human hydatid disease in high risk populations from central Tunisia. Preliminary results. Am J Trop Med Hyg.

[94] Mlika N, Larouzé B, Gaudebout C, Braham B, Allegue M, Dazza MC (1986). Echotomographic and serologic screening for hydatidosis in a Tunisian village. Am J Trop Med Hyg.

[95] Dada BJO (1980). Taeniasis, cysticercosis and echinococcosis/hydatidosis in Nigeria: I—prevalence of human taeniasis, cysticercosis and hydatidosis based on a retrospective analysis of hospital records. J Helminthol.

[96] Sixl W, Rosegger H, Schneeweiss H, Withalm H, Schuhmann G (1987). Serological investigations in Nigeria for anthropozoonoses in human sera: brucellosis, echinococcosis, toxoplasmosis, chlamydial diseases, listeriosis, rickettsiosis (*Coxiella burneti* and *Rickettsia conori*). J Hyg Epidemiol Microbiol Immunol.

[97] Kebede W, Hagos A, Girma Z, Lobago F (2009). Echinococcosis/hydatidosis: its prevalence, economic and public health significance in Tigray region, North Ethiopia. Trop Anim Health Prod.

[98] Koskei P, Janitschke K, Feseha G (2011). Prevalence of *Echinococcus granulosus* in some selected sites of Ethiopia. East Afr J Public Health.

[99] Mersie A (1993). Survey of echinococcosis in eastern Ethiopia. Vet Parasitol.

[100] Buishi I, Njoroge E, Zeyhle E, Rogan MT, Craig PS (2006). Canine echinococcosis in Turkana (north-western Kenya): a coproantigen survey in the previous hydatid-control area and an analysis of risk factors. Ann Trop Med Parasitol.

[101] Jenkins DJ, Gasser RB, Zeyhle E, Romig T, Macpherson CN (1990). Assessment of a serological test for the detection of *Echinococcus granulosus* infection in dogs in Kenya. Acta Trop.

[102] Macpherson CN, French CM, Stevenson P, Karstad L, Arundel JH (1985). Hydatid disease in the Turkana District of Kenya, IV. The prevalence of *Echinococcus granulosus* infections in dogs, and observations on the role of the dog in the lifestyle of the Turkana. Ann Trop Med Parasitol.

[103] Wachira TM, Sattran M, Zeyhle E, Njenga MK (1993). Intestinal helminths of public health importance in dogs in Nairobi. East Afr Med J.

[104] Inangolet FO, Biffa D, Opuda-Asibo J, Oloya J, Skjerve E (2010). Distribution and intensity of *Echinococcus granulosus* infections in dogs in Moroto District, Uganda. Trop Anim Health Prod.

[105] Oba P, Ejobi F, Omadang L, Chamai M, Okwi AL, Othieno E (2016). Prevalence and risk factors of *Echinococcus granulosus* infection in dogs in Moroto and Bukedea districts in Uganda. Trop Anim Health Prod.

[106] Nonaka N, Nakamura S, Inoue T, Oku Y, Katakura K, Matsumoto J (2011). Coprological survey of alimentary tract parasites in dogs from Zambia and evaluation of a coproantigen assay for canine echinococcosis. Ann Trop Med Parasitol.

[107] Kohil K, Benchikh El Fegoun MC, Gharbi M (2017). Prevalence of *Echinococcus granulosus* taeniasis in stray dogs in the region of Constantine (North-East Algeria). Bull Soc Pathol Exot.

[108] Awan MA, Gusbi AM, Beesley WN (1990). Echinococcosis in Libya. III. Further studies on the prevalence of *Echinococcus granulosus* in dogs. Ann Trop Med Parasitol.

[109] Ben Musa NA, Sadek GS (2007). Prevalence of echinococcosis in street dogs in Tripole District, Libya. J Egypt Soc Parasitol.

[110] Buishi IE, Njoroge EM, Bouamra O, Craig PS (2005). Canine echinococcosis in northwest Libya: assessment of coproantigen ELISA, and a survey of infection with analysis of risk-factors. Vet Parasitol.

[111] Gusbi AM (1987). Echinococcosis in Libya. I. Prevalence of *Echinococcus granulosus* in dogs with particular reference to the role of the dog in Libyan society. Ann Trop Med Parasitol.

[112] Packer DE, Ali TM (1986). *Echinococcus granulosus* in dogs in Libya. Ann Trop Med Parasitol.

[113] Amarir FE, Saadi A, Marcotty T, Rhalem A, Oukessou M, Sahibi H (2020). Cystic echinococcosis in three locations in the Middle Atlas, Morocco: estimation of the infection rate in the dog reservoir. Vector-Borne Zoonotic Dis.

[114] Dakkak A, El Berbri I, Petavy AF, Boue F, Bouslikhane M, Fassi Fihri O (2017). *Echinococcus granulosus* infection in dogs in Sidi Kacem Province (North West Morocco). Acta Trop.

[115] Pandey VS, Dakkak A, Elmamoune M (1987). Parasites of stray dogs in the Rabat region, Morocco. Ann Trop Med Parasitol.

[116] Pandey VS, Ouhelli H, Moumen A (1988). Epidemiology of hydatidosis/echinococcosis in Ouarzazate, the pre-Saharian region of Morocco. Ann Trop Med Parasitol.

[117] Saad MB, Magzoub M (1986). *Echinococcus granulosus* infection in dogs in Tambool, Sudan. J Helminthol.

[118] Bchir A, Jaiem A, Jemmali M, Rousset JJ, Gaudebout C, Larouze B (1987). Possible existence of an urban cycle of *Echinococcus granulosus* in central Tunisia. Trans R Soc Trop Med Hyg.

[119] Iraqi W (2017). Canine echinococcosis: the predominance of immature eggs in adult tapeworms of *Echinococcus granulosus* in stray dogs from Tunisia. J Helminthol.

[120] Lahmar S, Kilani M, Torgerson PR (2001). Frequency distributions of *Echinococcus granulosus* and other helminths in stray dogs in Tunisia. Ann Trop Med Parasitol.

[121] Lahmar S, Sarciron ME, Rouiss M, Mensi M (2008). *Echinococcus granulosus* and other intestinal helminths in semi-stray dogs in Tunisia: infection and re-infection rates. Tunis Med.

[122] Lahmar S, Boufana BS, Lahmar S, Inoubli S, Guadraoui M, Dhibi M (2009). *Echinococcus* in the wild carnivores and stray dogs of northern Tunisia: the results of a pilot survey. Ann Trop Med Parasitol.

[123] Verster A (1979). Gastro-intestinal helminths of domestic dogs in the Republic of South Africa. Onderstepoort J Vet Res.

[124] Adediran OA, Kolapo TU, Uwalaka EC (2014). *Echinococcus granulosus* prevalence in dogs in southwest Nigeria. J Parasitol Res.

[125] Arene FO (1984). Prevalence of toxocariasis and echinococcosis among dogs in the Niger Delta. J Trop Med Hyg.

[126] Awosanya EJ, Ligali Z, Duedu KO, Peruzzu A, Masala G, Bonelli P (2021). Prevalence of *Echinococcus granulosus* sensu lato in owned dogs in Lagos State, Nigeria. Vet Sci.

[127] Dada BJ, Adegboye DS, Mohammed AN (1979). A survey of gastro intestinal helminth parasites of stray dogs in Zaria, Nigeria. Vet Rec.

[128] Dada BJO (1980). Taeniasis, cysticercosis and echinococcosis/hydatidosis in Nigeria: IV—prevalence of *Echinococcus granulosus* infection in stray dogs. J Helminlhol.

[129] Karshima SN, Bata SI, Bot C, Kujul NB, Paman ND, Obalisa A (2020). Prevalence, seasonal and geographical distribution of parasitic diseases in dogs in Plateau State Nigeria: a 30-year retrospective study (1986–2015). J Parasit Dis.

[130] Okolo MI (1986). Prevalence and public health implications of *Echinococcus granulosus* in rural dogs in eastern Nigeria. Int J Zoonotic.

[131] Ugochukwu EI, Ejimadu KN (1985). Studies on the prevalence of gastro-intestinal helminths of dogs in Calabar, Nigeria. Int J Zoonotic.

